# Evaluation of the NS1 Rapid Test and the WHO Dengue Classification Schemes for Use as Bedside Diagnosis of Acute Dengue Fever in Adults

**DOI:** 10.4269/ajtmh.2011.10-0316

**Published:** 2011-02-04

**Authors:** Shera Chaterji, John Carson Allen, Angelia Chow, Yee-Sin Leo, Eng-Eong Ooi

**Affiliations:** Program in Emerging Infectious Diseases, Duke—National University of Singapore (NUS) Graduate Medical School, Singapore; Centre for Quantitative Medicine, Duke—NUS Graduate Medical School, Singapore; Communicable Diseases Centre, Tan Tock Seng Hospital, Singapore

## Abstract

Because healthcare facilities in many dengue endemic countries lack laboratory support, early dengue diagnosis must rely on either clinical recognition or a bedside diagnostic test. We evaluated the sensitivity and specificity of the 1997 and 2009 World Health Organization (WHO) dengue classification schemes and the NS1 strip test in acute sera from 154 virologically confirmed dengue patients and 200 patients with other febrile illnesses. Both WHO classification schemes had high sensitivity but lacked specificity. The NS1 strip test had high specificity, but its sensitivity was significantly lower in secondary compared with primary dengue infections. Differences in viral serotypes did not affect the performance of any of the three diagnostic approaches. Taken collectively, our findings indicate that the 1997 WHO dengue case definition can be used to exclude dengue, and the NS1 strip test can be used to confirm dengue infection, although the latter should be interpreted with caution in regions where secondary dengue infection is prevalent.

## Introduction

Dengue is an endemic, mosquito-borne viral disease throughout the tropics. Over 50 million dengue virus (DENV) infections are estimated to occur annually,^1^ and this number is projected to increase.^2^ Treatment is supportive with fluid replacement for plasma leakage, detected through regular monitoring for rising hematocrit levels, being the key feature.^3^ Although early diagnosis is useful in triaging patients, it could have a central role in dengue case management at a future time when antiviral drugs for dengue—the subject of intense research interest—become available for clinical use. In particular, immediate bedside diagnosis would be preferred to laboratory diagnosis, because the window of opportunity for antiviral therapy in dengue may be limited because of the short-lived viremia.^4^

The 1997 World Health Organization (WHO) dengue case definition^5^ has defined standards for diagnosis, clinical management, and reporting. Its main thrust was to enable disease classification for case management based on the presence of specific symptoms and signs. However, with the reemergence and global expansion of dengue, infection in adults has become increasingly common, and studies have found that the 1997 classification fails to detect a significant proportion of severe dengue cases in adults compared with children.^6^–^8^ These observations led to a revision of the WHO dengue case classification published in 2009.^9^ Although attention has been focused on the utility of these classification schemes in patient management,^6^–^8^ whether they can also be applied for early dengue diagnosis has not been evaluated, especially in adults.^10^

The recent availability of a rapid dipstick test, the Dengue NS1 Ag Strip (Bio-Rad Laboratories, Marnes-la-Coquette, France), that can provide results within 15 minutes could serve as a useful bedside diagnostic tool. NS1 is a highly conserved non-structural glycoprotein secreted by virus-infected cells during the acute phase of dengue,^11^,^12^ and it is essential for virus viability.^13^ However, it is not known how this test performs relative to the 1997 or 2009 WHO classification schemes.

Thus, the primary objective of this study was to compare the sensitivity and specificity of the NS1 strip with the 1997 and 2009 WHO classification schemes for the diagnosis of acute dengue fever. The secondary objectives were to evaluate the sensitivity of the tests in primary compared with secondary dengue infection, virus serotype, and clinical characteristics observed in the early stages of dengue illness.

## Materials and Methods

### Serum samples.

The Dengue NS1 Ag Strip was evaluated on archived serum samples collected from patients prospectively enrolled in the early dengue infection and outcome (EDEN) study.^14^ The EDEN study was approved by the National Healthcare Group Institutional Review Board (IRB) (DSRB B/05/013). Adult patients (18 years and above) presenting to various community polyclinics in Singapore within 72 hours of acute febrile illness (≥ 37.5°C) were enrolled with informed consent (first visit). Sera collected were tested for dengue virus using reverse transcription polymerase chain reaction (RT-PCR), virus isolation, and serology. A complete blood count was also performed on anticoagulated blood collected from all patients. The remaining serum was aliquoted and stored at −80°C until use. Sera were also collected from study participants at days 4–7 (second visit) and weeks 3–4 (third visit) after fever onset. Sera collected at the two later time points were also tested for DENV immunoglobulin M (IgM) and IgG antibodies.

A total of 1,811 patients have been enrolled to date in the EDEN study. Convenience sampling of the archived sera from dengue RT-PCR and/or virus isolation positive and negative was carried out. Altogether, sera from 354 patients were used in this study.

#### RT-PCR.

Viral RNAs were extracted using the QIAamp Viral RNA mini kit (Qiagen, Germany), and real-time RT-PCR was carried out using a set of primers targeting the 3′ non-coding region of dengue viruses.^15^ LightCycler software version 3.5 was used to analyze results. Positive control using RNA from the four DENV serotypes cultured in the *Aedes albopictus* mosquito (C6/36) cell line and water-only negative control were included in every RT-PCR run.

#### Virus isolation.

The C6/36 cell line (American Type Culture Collection [ATCC]: CRL-1660) was used for virus isolation. DENV antigens were detected using indirect immunofluorescence assay in conjunction with flavivirus, DENV group-specific, and DENV serotype-specific monoclonal antibodies from clarified hybridoma culture supernatant (ATCC: HB-46, -47, -48, -49, 112, and -114)^16^,^17^ and fluorescein isothiocyanate-conjugated goat anti-mouse antibody.

#### Serology.

Serum was tested for anti-DENV IgM and IgG antibodies using an IgM capture and indirect enzyme-linked immunosorbent assays (ELISAs), respectively (Panbio, Australia), according to the manufacturer's instructions.

### WHO dengue classification schemes.

Clinical data obtained from EDEN patients within 72 hours of fever onset were used in a diagnosis of probable dengue versus other febrile illness using the 1997 and 2009 WHO classification schemes. The 1997 WHO classification^5^ classifies acute febrile illness as dengue fever based on headache, retro-orbital pain, myalgia, arthralgia, rash, hemorrhagic manifestations, and leukopenia. In contrast, the 2009 WHO classification^9^ uses two or more clinical manifestations for probable dengue classification: nausea/vomiting, rash, aches and pains, tourniquet test positive, leucopenia, and any warning signs. For this analysis, leukopenia was defined as white blood cell (WBC) count below 4.5 × 10^3^/μL, and arthralgia/myalgia was included under aches and pains. Information on tourniquet test positivity was not obtained and hence, was not included in this analysis.

Because the goal of this study was to evaluate the performance of the dengue classification schemes as tools for early diagnosis, we have not included five of seven warning signs listed in the 2009 classification^9^ as criteria for diagnosis. These signs, namely clinical fluid accumulation, liver enlargement (> 2 cm), increase in hematocrit, persistent vomiting, and lethargy/restlessness, were present in some cases but at either the second visit or later, and they were detected through repeated monitoring. Hence, they may not be useful for early dengue diagnosis. Abdominal pain and mucosal bleeding were the only warning signs included in our diagnostic analysis.

### Immunochromatographic detection of dengue NS1 antigen.

The Dengue NS1 Ag Strip is a lateral flow immunochromatography test for the detection of the NS1 antigen. The manufacturer recommended that the NS1 Strip be read at 15 minutes and again at 30 minutes for doubtful samples or negative samples in patients with suggestive clinical information. In this study, the test was read at both 15 and 30 minutes for all serum samples, and the results were analyzed separately. Two technicians read each NS1 strip independently and were blinded to the infection status of the serum sample. If interpretations were conflicting, the final result was taken as equivocal.

### Statistics.

Statistical analysis was performed with SAS software, version 9.2. The two-sample *t* test was used to analyze continuous variables, and Fisher's exact test was used to compare binary variables and sensitivity.

## Results

Baseline demographic, clinical, and laboratory findings of the study populations are summarized in Table 1. Of the 354 sera, 153 were dengue positive by RT-PCR and virus isolation, and 1 serum sample was dengue positive by virus isolation alone. The 200 RT-PCR and virus isolation negative samples were also negative for antidengue IgM antibodies in the corresponding convalescent sera. These were classified as other febrile illness (OFI) for subsequent analyses. There was a slight male predominance in both dengue positive cases and other febrile illnesses, which is consistent with previous epidemiological observations in Singapore.^18^ Dengue positive patients were older than those with OFIs (39.4 versus 36.6 years, respectively; *P* = 0.040). Patients with OFIs sought medical care slightly earlier than patients with dengue (1.8 versus 2.1, respectively; *P* < 0.001). The WBC and platelet counts per microliter blood were significantly lower at first visit (baseline) for dengue cases than for OFIs (*P* < 0.001).

When the 1997 WHO dengue classification scheme was applied to the 354 total cases, 147 of 154 (95.4%) dengue cases and 128 (64.0%) of 200 OFI cases were diagnosed as dengue fever. Hence, the 1997 WHO classification scheme's sensitivity and specificity for dengue were 95.4% and 36.0%, respectively (Table 2). The 2009 WHO classification scheme identified 123 (79.9%) of dengue and 86 (43.0%) of OFI cases as probable dengue, giving sensitivity and specificity of 79.9% and 57.0%, respectively (Table 2). The NS1 strip had a sensitivity and specificity of 77.3% and 100%, respectively, at 15 minutes, and 80.5% and 100%, respectively, at 30 minutes (Table 2).

The comparative performance of the NS1 strip and 1997 and 2009 WHO classification schemes was statistically analyzed for dengue cases relative to presence or absence of pre-existing antidengue antibodies (IgG+/IgG−), DENV serotype (DENV-1, -2, or -3), and time from illness onset (day 1, 2, or 3). Clinical outcome in terms of hospitalization was also investigated. The data and analysis are summarized in Table 3. The WHO 1997 classification scheme identified most of the dengue cases in all categories analyzed. Statistically significant differences in sensitivity were observed between IgG+ and IgG− patients for the NS1 strip (*P* < 0.001) and the WHO 1997 classification scheme (*P* = 0.014) (Table 3).

When comparing the performance of the diagnostic approaches in primary and secondary infection, we took a positive finding on an antidengue IgG ELISA in the acute serum sample as indicative of secondary infection. Although antibodies to Japanese encephalitis virus (JEV; another flavivirus endemic in Southeast Asia) can cross-react with ELISA, Singapore has a very low seroprevalence of JEV.^19^ Table 3 shows that, consistent with previous observations, the NS1 strip had significantly higher sensitivity for primary infections (94.7%) than for secondary infections (67.1%; *P* < 0.001).^20^,^21^ The 1997 WHO classification had higher sensitivity for primary dengue (100%) than for secondary dengue (91.1%), and this difference was also statistically significant (*P* = 0.014). The 2009 WHO classification also had higher sensitivity for primary dengue (84.0%) than for secondary dengue (75.9%), but this difference was not statistically significant.

The sensitivity of the NS1 strip was lower in patients enrolled on day 1 of fever than those enrolled on days 2 and 3 (Table 3), and statistically significant differences in sensitivity were observed at 30 minutes (*P* = 0.010). However, the proportion of patients presenting with acute primary infection was lower on day 1 (35.9%, 14/39) compared with day 2 (60.3%, 35/58) and day 3 (45.6%, 26/57). On evaluating those with primary infections alone, no statistically significant difference was observed in the sensitivity of the NS1 strip for the first 3 days of febrile illness (at 30 minutes; *P* = 1.000). For secondary infections alone, there was an increase in sensitivity between sera collected on day 1 (44.0%) and that collected on day 3 (83.9%), and there were statistically significant differences at 30 minutes (day 1 versus day 3; *P* = 0.004). An increase in sensitivity of the 2009 WHO classification was also observed for patients who presented on day 3 (89.5%) compared with those who presented on day 1 (61.5%; *P* = 0.004) (Table 3).

No statistically significant differences were found in the sensitivity of the three diagnostic approaches in patients with different DENV serotypes (Table 3). A statistically significant difference was found for the 2009 WHO classification for patients who eventually received hospitalized care (86.0%) versus patients who were not hospitalized (72.1%; *P* = 0.043). No significant differences were found using the NS1 strip and the WHO 1997 classification scheme with regards to eventual hospitalization.

## Discussion

Medical centers in many dengue endemic regions lack ready access to a diagnostic laboratory that would enable early diagnosis of dengue. This lack of diagnostic resources portends an important rate-limiting obstacle in the proper usage of antiviral drugs after they become available. We have, therefore, focused this study on diagnostic approaches that can be carried out by the attending clinician without diagnostic laboratory support.

The performances of the WHO 1997 and 2009 classification schemes and the NS1 strip test were evaluated in serum samples that tested positive or negative using real-time RT-PCR and virus isolation for DENV. The highest sensitivity was observed in the 1997 classification (95.4%) followed by the NS1 strip (80.5%) and 2009 classification (79.9%). Conversely, specificity was highest in the NS1 strip (100%) followed by the 2009 and 1997 classifications (57.0% and 36.0%, respectively).

Although previous studies have described the clinical features that could potentially differentiate dengue from other acute febrile illnesses,^10^,^22^–^24^ these have not used the 1997 and 2009 classification schemes that are based primarily on a set of defined clinical symptoms and signs. Our findings suggest that the 1997 classification, with a sensitivity of 95.4%, could be very useful in ruling out dengue in the early febrile phase of illness, but confirmatory diagnosis cannot be achieved given the poor specificity.

An obvious solution to dengue diagnosis is, thus, to use the 1997 classification scheme to rule out dengue and to use a bedside diagnostic test such as the NS1 strip to confirm the diagnosis. However, the performance of the latter has some important limitations. Compared with primary infections, the NS1 strip has lower sensitivity for secondary infections where pre-existing NS1 antibodies in the serum could inhibit the detection of NS1 antigen.^20^,^21^,^25^–^27^ Hence, for patient populations in dengue hyperendemic countries where multiple DENV serotypes cocirculate, the NS1 strip might not be adequate for bedside confirmation of dengue diagnosis.

An important question is whether the different approaches to dengue diagnosis would miss those that eventually developed severe disease. In the 2009 classification, severe dengue disease includes those with plasma leakage, internal hemorrhage, or severe organ impairment. Unfortunately, the number of cases that presented with these clinical outcomes was small, and a meaningful analysis could not be conducted. We have instead tested for differences in hospitalization rate among those identified as dengue using different diagnostic approaches and have found a statistically significant difference only for the 2009 WHO classification scheme.

The NS1 strip also seemed to be less sensitive when used in patients within the first day from illness onset. In our collection of samples, a substantial proportion of patients with secondary dengue infection (DENV IgG positive) presented on the first day of fever for reasons yet to be determined. This resulted in reduced sensitivity of the NS1 strip on day 1 compared with days 2 and 3 of illness. On evaluating the sensitivity of the NS1 strip for primary and secondary infections versus day of illness, the findings of this study agree with another report,^27^ which found an increase in sensitivity of the NS1 strip for secondary infections between days 0 and 3.

Reports on the sensitivity of the NS1 strip for the four dengue virus serotypes have varied depending on the geographical region of testing. Earlier reports found no significant difference in sensitivity of the NS1 strip for different serotypes^27^; however, recent studies from Vietnam^21^ and Venezuela^25^ have found that the sensitivity of the NS1 strip was lower for DENV-2 infections. A possible explanation for this finding is that a clinically overt DENV-2 infection is more likely to occur in the context of secondary infection than primary infection and hence, the associated reduction in sensitivity of the NS1 strip.^21^ In this study, no statistically significant differences were observed between the serotypes when divided into primary or secondary infections.

The strength of this case-control study is the use of serum obtained from prospectively enrolled cases with standardized collection of clinical information. Laboratory tests were used to confirm the presence or absence of acute dengue infection in both cases and controls. All the patients presented with acute febrile illness within the first 72 hours from fever onset. This period represents the viremic phase and is relevant to eventual antiviral applications. The main limitation of this study is that, for the 2009 classification, not all warning signs could be included for analysis. A tourniquet test was not carried out.^14^ However, the results of this test in making diagnoses are mixed^7^,^22^ and would not be expected to substantially alter our findings. Interpretation of what constitutes persistent vomiting and lethargy/restlessness proved difficult from our clinical data obtained through a standardized questionnaire, and we have, thus, not included these for analysis. This study is also limited to adult patients, and the sensitivity and specificity of the WHO classification schemes may differ in younger age groups.^28^ Furthermore, the majority of our population was of Chinese ethnicity, and the performance of the NS1 could differ in different races or in viruses circulating in different regions of the world.^29^

In conclusion, the 1997 WHO dengue case definition can be useful in ruling out dengue, whereas the Dengue NS1 Ag Strip can be used as a bedside diagnostic test to support a diagnosis of acute dengue, although caution is advised in the interpretation of this test in dengue hyperendemic areas.

## Figures and Tables

**Table 1:** Baseline demographic, hematological, clinical, and virological information of the study population

	Dengue (*N* = 154)	OFI (*N* = 200)	*P* value*
Demographics			
Male gender (%)	89 (57.8)	121 (60.5)	0.700
Age in years (SD)	39.4 (13.4)	36.6 (13.2)	0.040
Chinese ethnicity (%)†	119 (77.3)	133 (66.5)	0.030
Days from illness onset at enrollment (SD)	2.1 (0.8)	1.8 (0.8)	< 0.001
Clinical presentation			
Headache (%)	126 (81.8)	132 (66.0)	0.001
Retro-orbital pain (%)	41 (26.6)	27 (13.5)	0.003
Myalgia (%)	106 (68.8)	123 (61.5)	0.200
Arthralgia (%)	96 (62.3)	107 (53.5)	0.100
Rash (%)	16 (10.4)	6 (3.0)	0.007
Nausea (%)	79 (51.3)	61 (30.5)	< 0.001
Vomiting (%)	25 (16.2)	17 (8.5)	0.030
Mucosal bleed (%)	11 (7.1)	6 (3.0)	0.080
Abdominal pain (%)	12 (7.8)	43 (21.5)	< 0.001
Leukopenia < 4.5 × 10^3^/μL (%)	111 (72.1)	23 (11.5)	< 0.001
Thrombocytopenia < 100 × 10^3^/μL (%)	23 (14.9)	3 (1.5)	< 0.001
DENV serotype			
DENV-1 (%)	54 (35.1)		
DENV-2 (%)	46 (29.9)		
DENV-3 (%)	54 (35.1)		
DENV-4 (%)	0		
Serological status			
Primary infection (%)	75 (48.7)		
Secondary infection (%)	79 (51.3)		

* Continuous variables, *t* test; categorical variables, Fisher's exact test.

† Other ethnic groups in the study population were Malays, Indians, and Caucasians.

**Table 2:** Sensitivity and specificity of the Dengue NS1 Ag Strip test and 1997 and 2009 WHO classification schemes relative to RT-PCR and virus isolation

Diagnostic modality	Sensitivity (95% CI)*	Specificity (95% CI)†
Dengue NS1 Strip 15 minutes	77.3% (69.8–83.6)	100% (98.5)‡
Dengue NS1 Strip 30 minutes	80.5% (73.5–86.5)	100% (98.5)‡
WHO 1997	95.4% (90.9–98.2)	36.0% (29.4–43.1)
WHO 2009	79.9% (72.7–85.9)	57.0% (49.8–64.0)

* *N* = 154.

† *N* = 200.

‡ Lower one-sided (exact).

**Table 3:** The performance of the NS1 strip and 1997 and 2009 WHO classification schemes in secondary DENV infection, infection with different DENV serotypes, time of clinical presentation, and outcome in terms of hospitalization

Variable	Total dengue (%)*	Number (%) testing positive		
		NS1 (*N* = 124)	WHO 1997 (*N* = 147)	WHO 2009 (*N* = 123)
Serological status at first visit				
IgG+ (secondary infection)	79 (51.3)	53 (67.1)	72 (91.1)	60 (75.9)
IgG— (primary infection)	75 (48.7)	71 (94.7)	75 (100)	63 (84.0)
*P* value†		< 0.001	0.014	0.234
DENV serotype				
DENV-1	54 (35.1)	43 (79.6)	53 (98.1)	44 (81.5)
DENV-2	46 (29.9)	34 (73.9)	43 (93.5)	35 (76.1)
DENV-3	54 (35.1)	47 (87.0)	51 (94.4)	44 (81.5)
*P* value†		0.256	0.508	0.793
Day of fever				
1	39 (25.3)	25 (64.1)	36 (92.3)	24 (61.5)
2	58 (37.7)	48 (82.8)	56 (96.6)	48 (82.8)
3	57 (37.0)	51 (89.5)	55 (96.5)	51 (89.5)
*P* value†		0.010‡	0.551	0.004§
Clinical outcome				
Hospitalized	86 (55.8)	73 (84.9)	82 (95.3)	74 (86.0)
Not hospitalized	68 (44.2)	51 (75.0)	65 (95.6)	49 (72.1)
*P* value†		0.153	1.000	0.043

* *N* = 154.

† Fisher's exact test.

‡ Day 1 vs. 2, *P* = 0.054; day 1 vs. 3, *P* = 0.004; day 2 vs. 3, *P* = 0.420.

§ Day 1 vs. 2, *P* = 0.032; day 1 vs. 3, *P* = 0.002; day 2 vs. 3, *P* = 0.420.
